# Study of the Microfocus X-Ray Tube Based on a Point-Like Target Used for Micro-Computed Tomography

**DOI:** 10.1371/journal.pone.0156224

**Published:** 2016-06-01

**Authors:** Rifeng Zhou, Xiaojian Zhou, Xiaobin Li, Yufang Cai, Fenglin Liu

**Affiliations:** 1 The Key Lab for Opto-electronic Technology & Systems of the Education Ministry of China, ICT Research Center, University of Chongqing, Chongqing, China; 2 Engineering Research Center of ICT Nondestructive Testing of the Education Ministry of China, University of Chongqing, Chongqing, China; 3 Nuclear and radiation safe center, Ministry of Environmental Protection of People’s Republic of China, Beijing, China; North Shore Long Island Jewish Health System, UNITED STATES

## Abstract

For a micro-Computed Tomography (Micro-CT) system, the microfocus X-ray tube is an essential component because the spatial resolution of CT images, in theory, is mainly determined by the size and stability of the X-ray focal spot of the microfocus X-ray tube. However, many factors, including voltage fluctuations, mechanical vibrations, and temperature changes, can cause the size and the stability of the X-ray focal spot to degrade. A new microfocus X-ray tube based on a point-like micro-target in which the X-ray target is irradiated with an unfocused electron beam was investigated. EGS4 Monte Carlo simulation code was employed for the calculation of the X-ray intensity produced from the point-like micro-target and the substrate. The effects of several arrangements of the target material, target and beam size were studied. The simulation results demonstrated that if the intensity of X-rays generated at the point-like target is greater than half of the X-ray intensity produced on the substrate, the X-ray focal spot is determined in part by the point-like target rather than by the electron beam in the conventional X-ray tube. In theory, since it is able to reduce those unfavorable effects such as the electron beam trajectory swinging and the beam size changing for the microfocus X-ray tube, it could alleviate CT image artifacts caused by the X-ray focal spot shift and size change.

## Introduction

**Fig 1:**
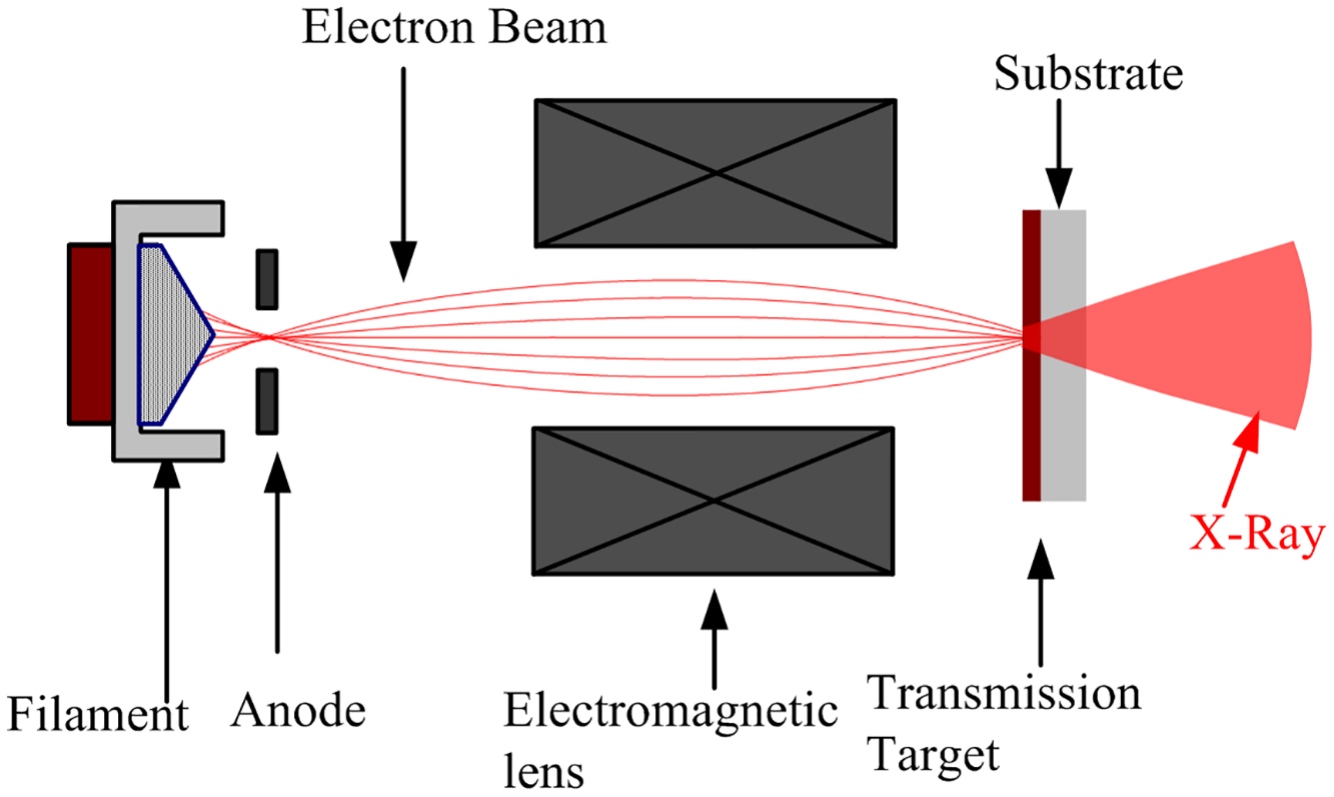
Diagrammatic sketch of a conventional microfocus tube with a transmission target.

In recent years, X-ray micro-computed tomography (Micro-CT) or even Nano-CT systems have become capable of preclinical studies to provide structural information, due to their ability to achieve quantification of internal structures at submicron-to-nanometer scales. These tools are routine in many research domains such as life science, material science, environmental science, energy science, etc. [1,2].The microfocus X-ray tube is one of the key components of Micro-CT devices because the spatial resolution of the CT image is, for the most part, decided by the effective focal spot size of the X-ray tube[3,4]. A conventional microfocus X-ray tube is composed of a cathode (filament), an anode, an electromagnetic lens, and a target, as shown in Fig 1. The electron beam emanated from the cathode is accelerated by an electric field between the cathode and the anode, and impacts on the transmission target where the X-rays are generated [5]. The effective focal spot size and the position stability of the X-rays beam are determined by the size and accelerating trajectory of the electron beam in the tube. To minimize the active X-ray focal spot size, a well-focused and stable electron beam trajectory is required [6]. However, many factors can influence the size and the trajectory stability of electron beams, in fact, such as voltage fluctuations, mechanical vibrations and temperature changes. For example, in a Micro-CT system using a tube with an effective focus spot size of approximately 5μm, the shift of the X-ray focal spot is more than 35μm, caused by electron beam drift after 20 min of radiation time. This is considered to be a main factor leading to motion artifacts in micro-CT images [7,8]. These motion artifacts are quite difficult to eliminate through general correction methods [9,10].

Ihsan *et al*. [11] proposed a micro-structured X-ray target for the purpose of achieving a small active X-ray focal spot size with an unfocused electron beam. They assumed that X-rays are produced only at the micro-structured target when irradiated with a larger scale electron beam, so the focal spot size of the generated X-ray is determined only by the lateral size of the microstructures. In fact, many factors including the thickness of the substrate, the size of the electron beam, the material of the micro-target and the substrate were not taken into account in those references. For instance, when the thickness of the substrate is greater than the electron’s range in the substrate material, and a significantly larger region of the substrate becomes under direct irradiation by an electron beam, a large number of X-rays are emanated from the substrate. Due to the contributions of these photons, the effective focal spot size could be enlarged. Therefore, the X-ray focal spot size is determined by the incident electron beam rather than by the micro-structured target.

**Fig 2:**
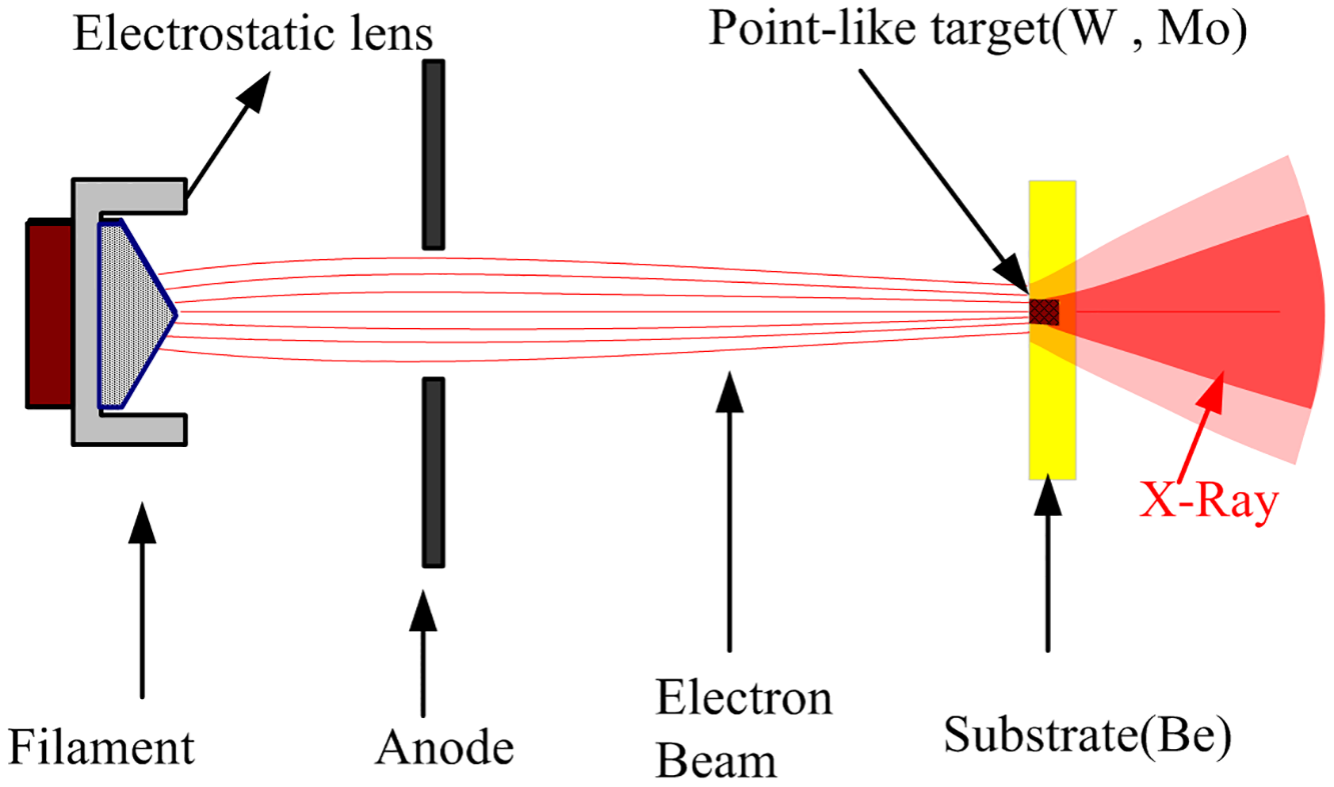
Diagrammatic sketch of a novel microfocus tube with a point-like micro-target.

To solve these problems, as illustrated in Fig 2, we proposed a point-like micro-target made of a high atomic number (Z) metal like tungsten (W) or molybdenum (Mo) attached to a substrate manufactured using a low atomic number material such as beryllium(Be) or diamond as an X-ray target (anode). In this paper, we illustrated that this novel target may achieve an X-ray focal spot with not only a micrometer size but also with superior position stability for the micro-CT system. The factors which primarily influenced the focal spot size—including the height of the point-like target—and various ratios of micro-target X-rays to substrate X-rays were investigated in detail via Monte Carlo (MC) simulation, and the optimum dimensions of the point-like micro-target were proposed. Degradation of CT image quality has been studied for various ratios of target X-rays to substrate X-rays. As a result, when the point-like micro-target and target substrate is designed optimally, the effective X-ray focal spot size of this tube is determined, in part, only by the size of the point-like micro-target instead of by the size of the electron beam in the traditional X-ray tube. On the other hand, due to the independence of the electron beam trajectory, the X-ray focal spot would remain stable.

Besides, since the massive and complex electromagnetic lens is not required for this microfocus X-ray tube, the volume could be reduced remarkably, which is significant in order to miniaturize the micro-CT system.

## Theories and Methods

### 1. Bremsstrahlung Radiation

The accelerated electrons in the tube can be slowed down through interactions with nuclei in the target, leading to the emission of bremsstrahlung photons. The conversion efficiency of electrons and bremsstrahlung photons has been expressed in numerous previous publications [12–14].

To describe continuum tube radiation irradiated from thin targets, the semiempirical formula, which is a well-known method proposed and developed by Kramers and Ambrose *et al*. [15–17], is employed. This method is used to express the intensity of X-ray photons Φ_*c*_(*x*,*hv*) emitted from the position *x* of target within the energy interval from *hv* to *hv* + *d*(*hv*) as:

$$\Phi_{c}\left(x,h\nu \right)=Ki_{e}Z^{n}{\left(\frac{h\nu_{0}}{h\nu }-1\right)}^{x}\Omega f$$ (1)

where Z is the atomic number of the target material; *K* is the constant *K* = 1.35 × 10^9^ *photons sr*^−1^*mA*^−1^*keV*^−1^*s*^−1^; *hv*_*0*_ the maximum photon energy equal to the operation voltage; *i*_*e*_ the tube current; Ω the solid angle in which the X-ray photons are emitted; *n* = 1, *x* = 1.109 − 0.00435*Z* + 0.00175*hv*_0_, and *f* the absorption term which describes the attenuation of emitted X-rays into the anode by Love and Scott’s distribution [11,18].

(Eq 1) shows that the intensity of X-ray photons Φ(*x*,*hv*) produced by the electron beam impacting on the micro-target has heavily depended on atomic number Z. It is obvious that the intensity of X-ray photons Φ(*x*,*hv*) produced on higher Z materials is much more than those generated on the low Z materials if the electron energy (keV) is appropriate.

### 2. Material and Structure

**Fig 3:**
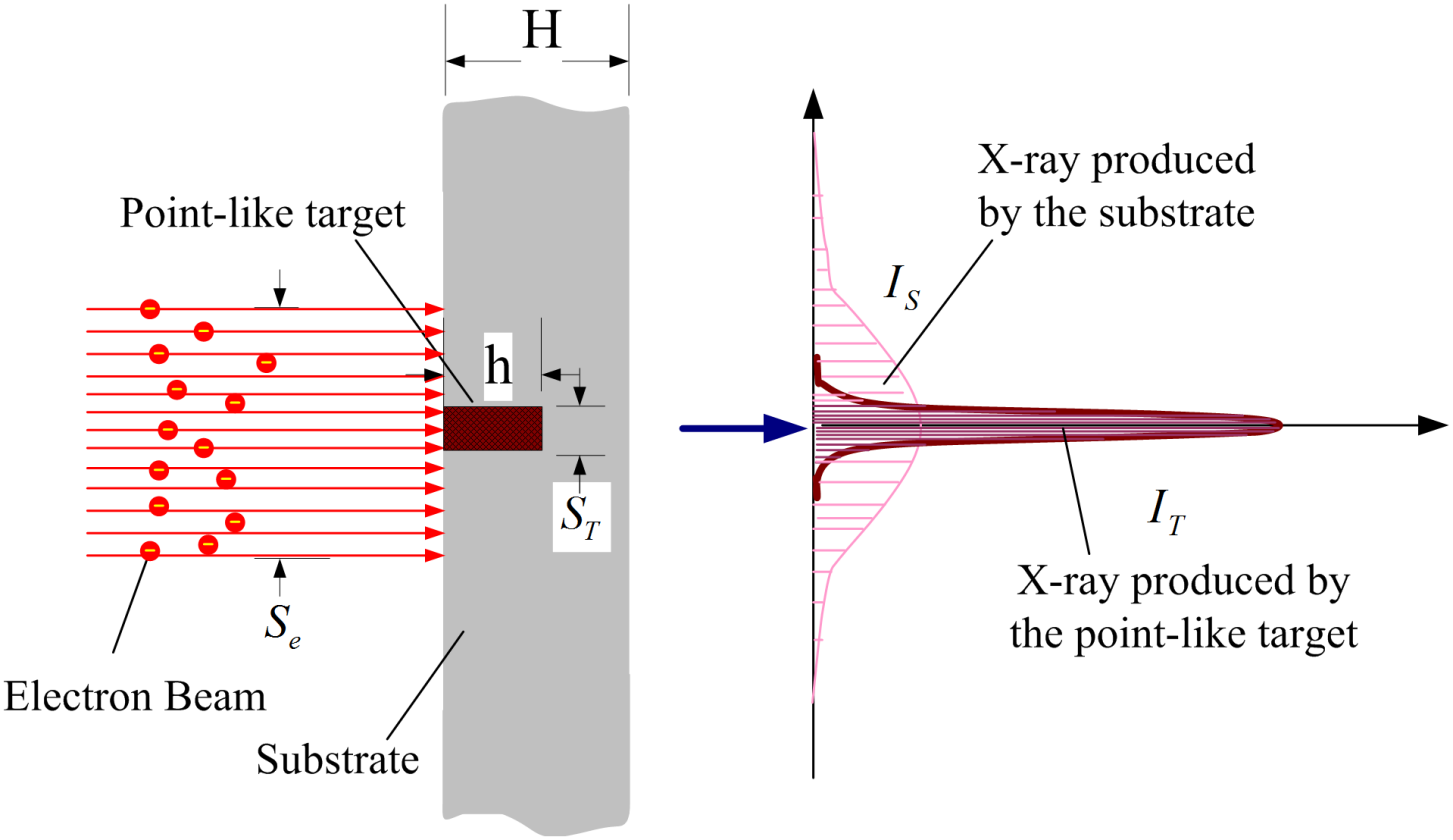
Model of a point-like micro-target. The left curves indicate the difference of the X-ray intensity produced by the point target and that by the substrate.

The novel micro-target, as shown in Fig 3, is consisted of a point-like micro-target made of high Z metals materials (W,Mo.*et al*.) combined with a low Z substrate like Be or diamond. In particular, the diameter of the point-like micro-target may be less than that of the incident electron beam. Dimensions of the point-like micro-target and target substrate are shown in Table 1.

**Table 1:** The dimension of the point-like micro-target and target substrate.

	Name	Symbol	Size(μm)
1	Diameter of point-like micro-target	S_T_	Φ = 0.5~5.0 (cylinder)
2	Height of point-like micro-target	h	1.0~5.0
3	Thickness of target substrate	H	250~500
4	Diameter of electron beam	S_e_	Φ = 1.0~10.0 (cylinder)

As shown in Fig 3, the diameter of the electron beam and the point-like micro-target is *S*_*e*_,*S*_*T*_ respectively. If *S*_*e*_ > *S*_*T*_, the bremsstrahlung radiation will be sent from the point-like micro-target, as well as from the target substrate. In solid angle *d*Ω_0_,*I*_*T*_ is the intensity (total fluence/unit incident electron fluence) of X-ray produced by the point-like micro-target, and *I*_*s*_ is the intensity of X-ray generated by the target substrate, defined respectively as:

$$I_{T}=\int_{0}^{h\nu_{0}}\int_{S_{T}}^{}\Phi_{T}(x,h\nu )d(h\nu )ds$$ (2)

$$I_{S}=\int_{0}^{h\nu_{0}}\int_{S_{e}-S_{T}}^{}\Phi_{S}(x,h\nu )d(h\nu )ds$$ (3)

Where *hv*_0_ *= U*_*e*_ is the maximum energy of the X-ray corresponding to the maximum energy of the incident electron (keV), Φ_*T*_(*x*,*hv*) and Φ_*S*_(*x*,*hv*) denote the photon’s fluence produced by the point-like micro-target and that produced by the target substrate within the energy interval from *hv* to *hv*+*d*(*hv*), respectively.

In theory, the effective X-ray focal spot size is determined by the cooperation of the bremsstrahlung radiation *I*_*T*_ and *I*_*S*_. For instance, if *I*_*T*_<<*I*_*S*_, the size of the effective X-ray focal spot would almost be determined by the electron beam size. However, when *I*_*T*_>>*I*_*S*_,that to say, when the production of the X-rays on the substrate is far fewer than that of the X-rays on the point-like micro-target, the adverse effects on the radiation imaging by the photons produced in the target substrate could be neglected. Therefore, the effective X-ray focal spot size may be determined by the point-like micro-target rather than determined by the electron beam. Therefore, if *Z*, *hv*,*S*_*T*_,*S*_*e*_,*h* and *H* are chosen reasonably, as indicated in Fig 3, the difference of the intensity distribution curves of *I*_*T*_ and *I*_*S*_ is quite significant.*I*_*T*_ / *I*_*S*_ defined as:

ITIS=∫0hν0∫STΦT(x,hν)d(hν)ds∫0hν0∫Se−STΦS(x,hν)d(hν)ds (4)

Obviously, *I*_*T*_ / *I*_*S*_ is a key factor that affects X-ray focal spot size in this new type of X-ray tube. In the following sections, we will put emphasis on the calculation and simulation of the parameters including *Z*, *hv*, *S*_*T*_ / *S*_*e*_,*h* and *H*, which are the main factors affecting the *I*_*T*_ / *I*_*S*_.

## Simulation Methods and Results

### 1. Simulation of *I*_*T*_ / *I*_*S*_

#### Simulation tool and methods

Nowadays, it is acknowledged that the Monte Carlo method is a well-established and highly effective approach for simulating the transport of electron and radiation in materials [19–21]. During this research, an enhanced EGS4 code—which is especially suited to simulate the coupled transport of electrons, positrons and photons in materials, with the accuracy of simulation results better than 10% for energies of charged particles down to 10 keV and photons down to 1 keV [22–24]—was implemented to calculate the X-ray’s intensity and the X-ray’s spectral distribution of the point-like micro-target with different target thicknesses, substrates and tube voltages. We believe that the codes were reliable enough for our purposes.

As described in the previous section, calculations for *I*_*T*_ / *I*_*S*_ involve several mutually contending and interrelating parameters: materials of point-like micro-target and target substrate, the height of point-like micro-target *h* and the height of target substrate *H*, and the ratio of the diameter of point-like micro-target to the diameter of the electron beam *S*_*T*_ / *S*_*e*_. There is always a mutual trade-off among these parameters.

#### The influence of *h* of different point-like target materials (W, Mo, Cu) on *I*_*T*_ / *I*_*S*_

**Fig 4:**
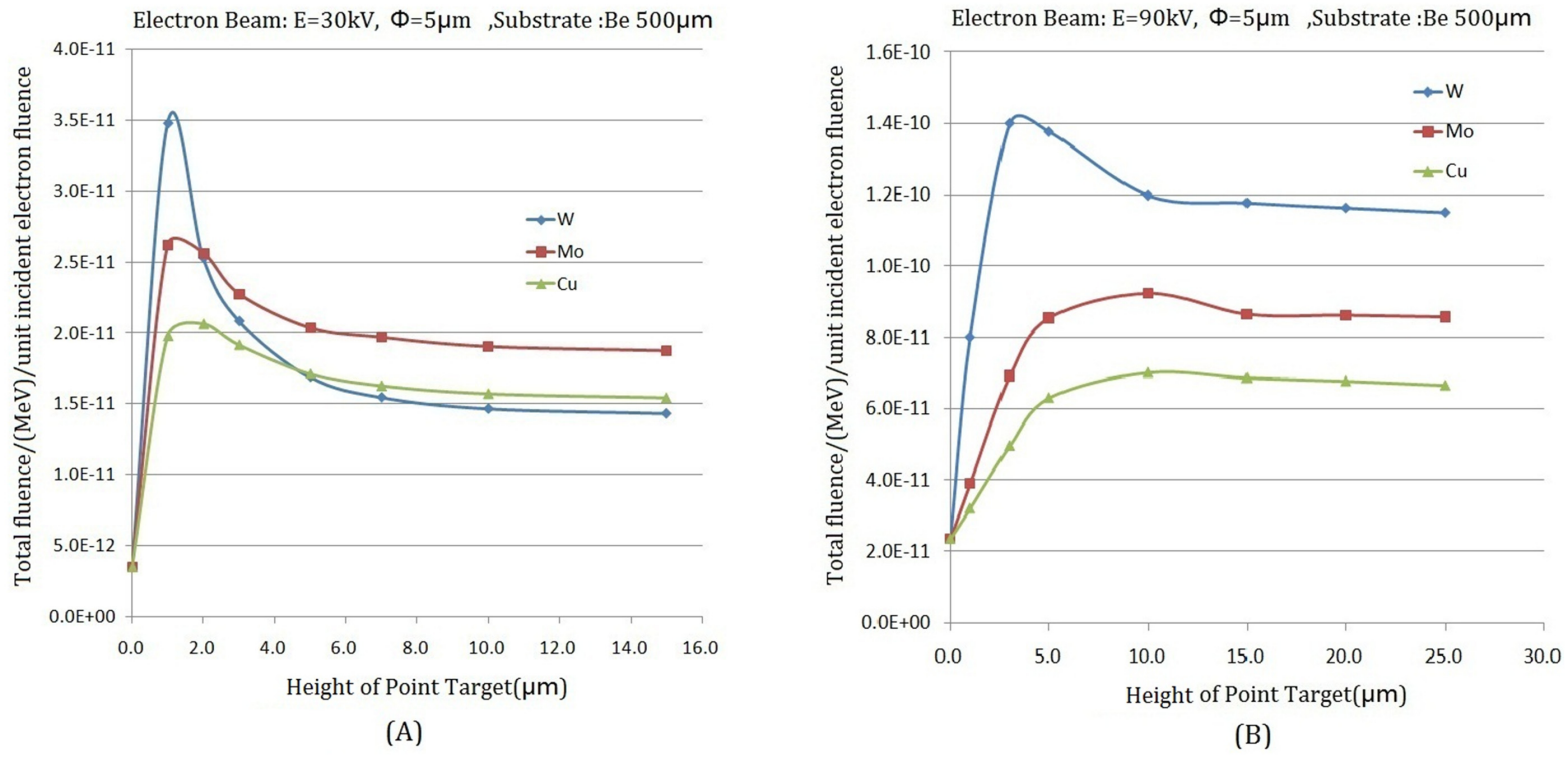
The relevance of the intensity of X-ray on the height h of a point-like micro-target with several materials (W, Mo, Cu). (A) electron beam energy 30keV; (B) electron beam energy 90keV.

In a transmission target, with the thickness of target increased, X-ray attenuation becomes enhanced during the event of X-rays passing through the target. So it is obvious that the target thickness has a significant effect on the intensity of X-ray radiation. The dependence of the point-like micro-target height *h* on the intensity of the X-rays generated by three material targets (W,Mo,Cu) was investigated with the enhanced EGS4 code. The beam energy, in this case, was 30 keV and 90 keV; the thickness of the target substrate beryllium (Be) is 500μm; and the diameter size of the incident electron beam and the point-like micro-target are all 5.0μm. The simulation results revealed that the X-ray intensity (total fluence/unit incident electron fluence) increases with the increasing height of the point-like micro-target, as shown in Fig 4. However, the X-ray intensity will reduce when raising the height further after reaching a maximum value of all materials. As displayed in Fig 4(A) (*E*_*e*_ = 30*keV*), the maximum of X-ray intensity which is produced by the point-like micro-target (W) with 1.0μm height is 10 times more than that produced on the target substrate (i.e. the point where the height of point target is zero), i.e. *I*_*T*_ / *I*_*S*_ > 10.0. When changing the energy of the incident electron beam, as displayed in Fig 4(B) (*E*_*e*_ = 90*keV*), the dependence of *I*_*T*_ and *h* is the same as with low energy. The maximum of X-ray intensity is produced by W point-like micro-target with2.0μm, i.e. *I*_*T*_ / *I*_*S*_ >6.0. The simulation results show that *I*_*T*_ / *I*_*S*_ becomes decreased with the increasing energy of the incident electron beam.

#### The influence of the X-ray spectral distribution on *I*_*T*_ / *I*_*S*_

**Fig 5:**
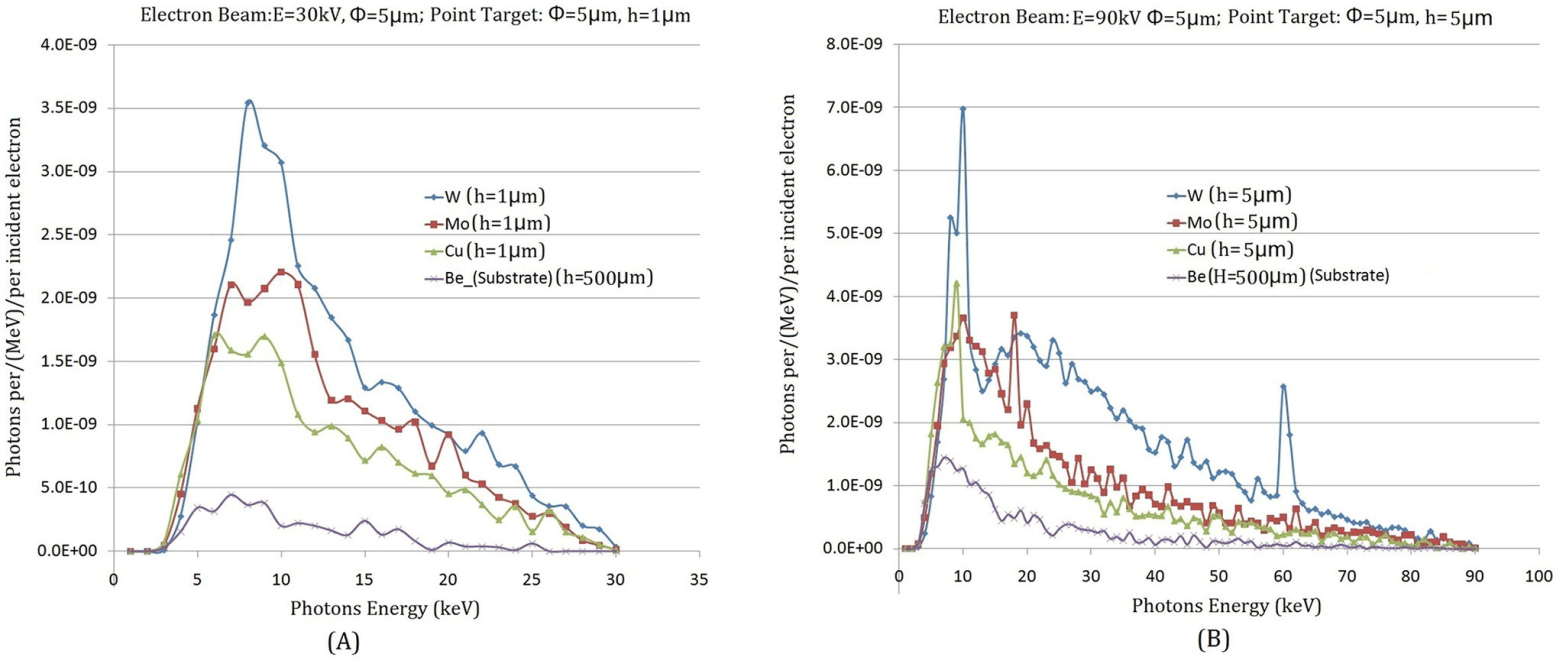
X-ray spectral distributions of point-like micro-targets irradiated by some different materials. (A) electron beam energy 30keV, height of point-like micro-targets is 1.0μm; (B) electron beam energy 90keV, height of point-like micro-targets is 5.0μm.

Several X-ray spectral distributions produced by different materials(W,Mo,Cu) with point-like micro-targets with diameter φ = 5.0μm,height *h* = 1.0μm, and with the incident electron beam energy 30keV, are shown in Fig 5(A); micro-targets with height *h* = 5.0μm, and the incident electron beam energy 90keV are shown in Fig 5(B), respectively. The simulation results illuminated that the differences between the X-ray spectral distribution generated by point-like micro-targets and that generated by target substrate are quite distinct. Compared to the average energy of photons generated by the point-like micro-target, the average energy of photons generated by the target substrate is lower distinctly. In practice, if choosing an appropriate filter placed in front of the window of the tube, it is easy to reduce those low energy photons generated by the target substrate, thus leading to the increasing of *I*_*T*_ / *I*_*S*_, which will obviously benefit CT image resolution.

#### The relevance of *S*_*T*_ / *S*_*e*_ to *I*_*T*_ / *I*_*S*_

**Fig 6:**
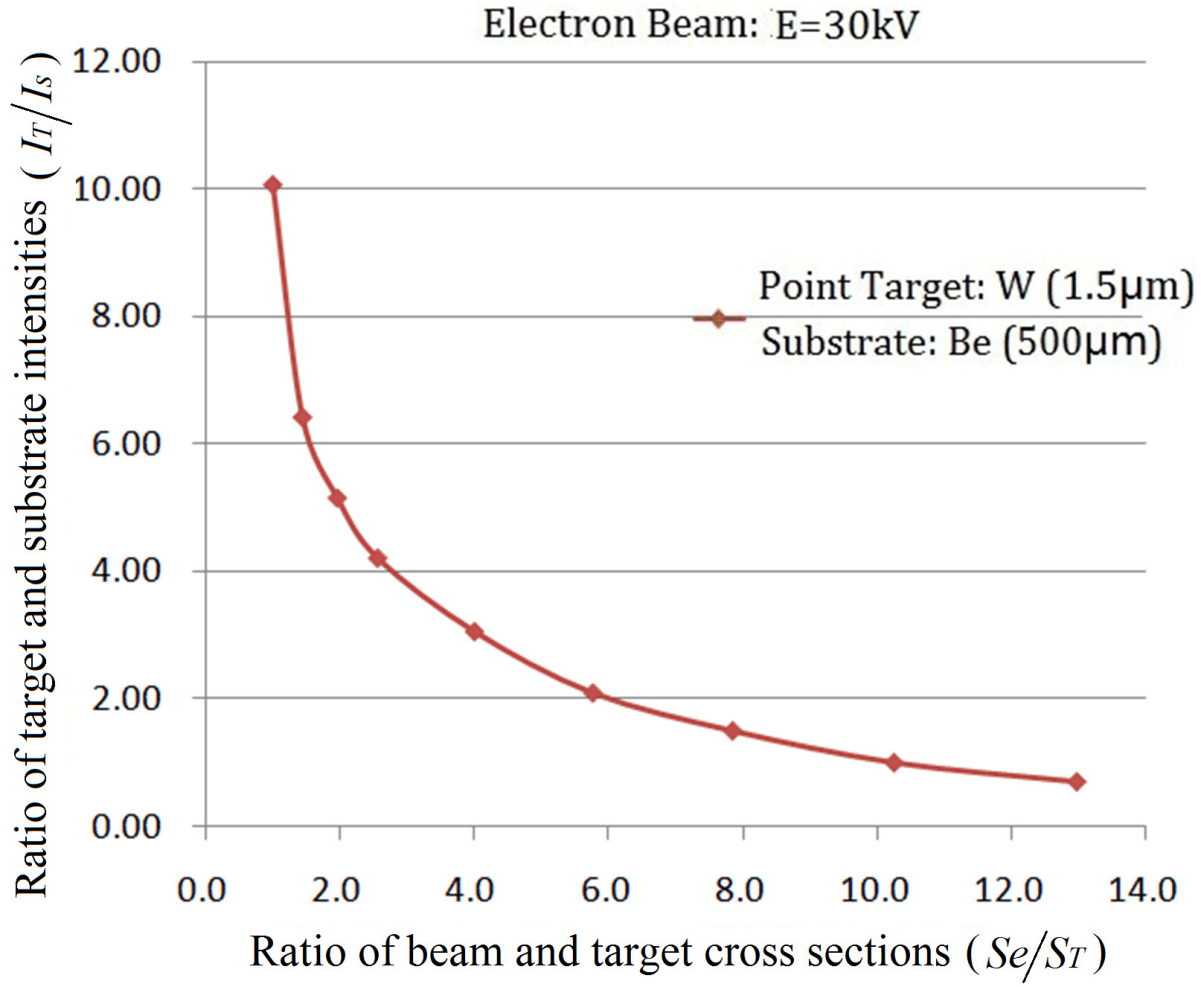
Relevance of the ratio of the X-ray intensity produced by the point-like micro-target to the X-ray intensity produced by the target substrate *I*_*T*_ / *I*_*S*_ with the ratio of the diameter of point-like micro-target to the diameter of electron beam *S*_*e*_ / *S*_*T*_.

To show the importance of *S*_*T*_ / *S*_*e*_ to *I*_*T*_ / *I*_*S*_, as described in Eq 4, the simulation was implemented with geometry shown in Fig 3: the height of the point-like micro-target 1.0μm, the thickness of the target substrate 500μm, and with the electron beam energy 30keV. In Fig 6, the horizontal ordinate represents the ratio of the diameter of the point-like micro-target to the diameter of the electron beam *S*_*T*_ / *S*_*e*_, and the vertical ordinate denotes the ratio of the intensity of X-rays produced by the point-like micro-target to the target substrate *I*_*T*_ / *I*_*S*_. The relevance of the curve of *I*_*T*_ / *I*_*S*_ to *S*_*T*_ / *S*_*e*_ in Fig 6 displays that the *I*_*T*_ / *I*_*S*_ decreases with *S*_*T*_ / *S*_*e*_ increasing rapidly. When *S*_*T*_ / *S*_*e*_ = 1/2, then *I*_*T*_ / *I*_*S*_>5.0; When *S*_*T*_ / *S*_*e*_ = 1/13, then *I*_*T*_ / *I*_*S*_>1/2.

#### Effect of the thickness of the target substrate on *I*_*T*_ / *I*_*S*_

**Fig 7:**
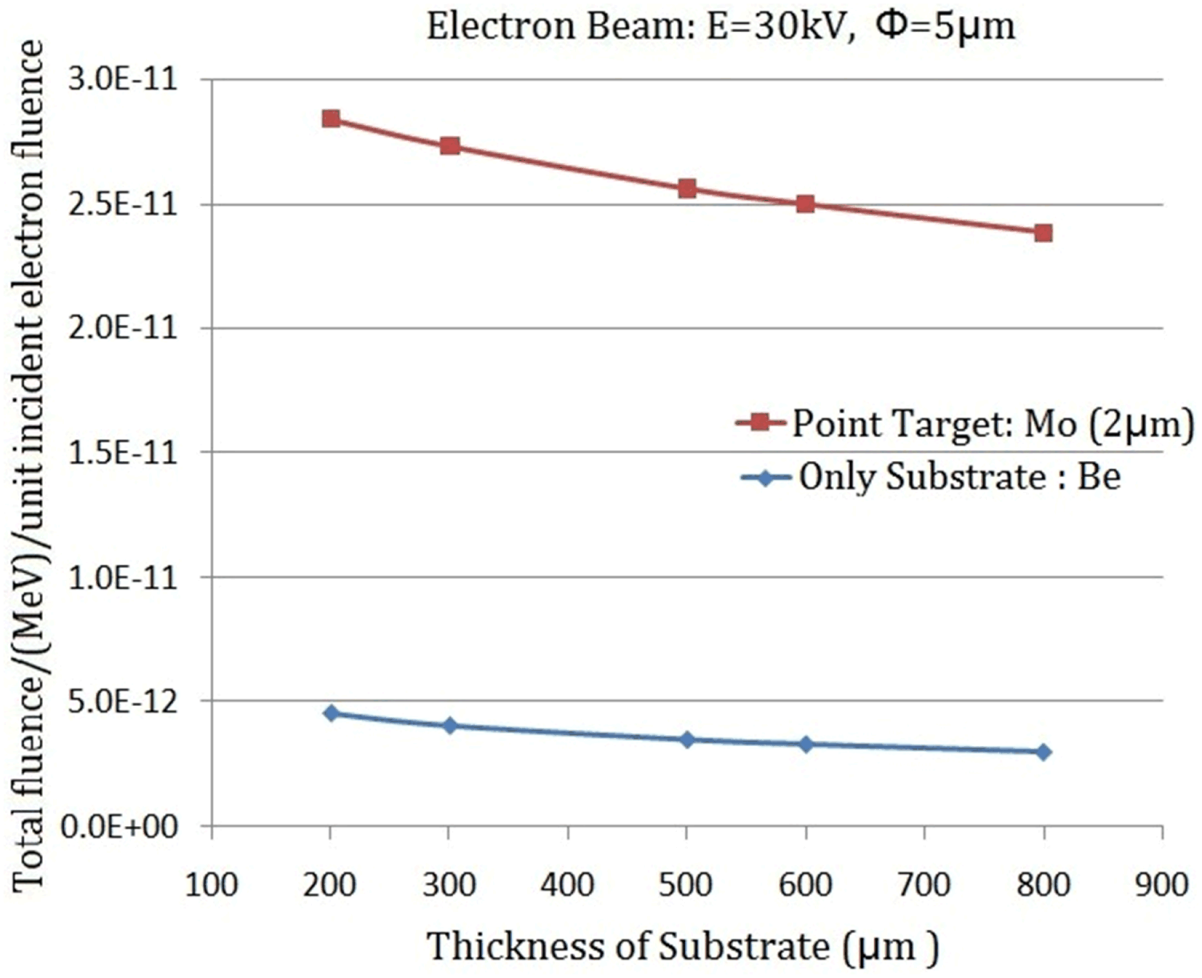
Effect of the thickness of the target substrate on the ratio of X-ray intensity *I*_*T*_ / *I*_*S*_.

Generally, the target substrate not only acts as a substrate for the point-like micro-target but also as the window of the X-ray tube. Hence, on the one hand, to withstand the air pressure external the tube, the thickness of the substrate is required to ensure its mechanical strength. On the other hand, if the thickness is too heavy, it will attenuate the photons severely during the penetration of X-ray through the substrate. Low Z materials with substantial mechanical strength like beryllium (Be) or diamond, etc. are used as the substrate materials. To study the effect of thickness of the substrate on the intensity of the X-rays, the simulation experiment was implemented under the condition of electron beam energy 30keV, with the thickness of the substrate ranging from 200μm to 800μm.The results are shown in Fig 7 and present that with and without a point-like micro-target, the intensity of X-ray decreased with the increasing thickness of the substrate slightly. For instance, when increasing the thickness of the substrate from 500μm to 800μm, the X-ray intensity decreased by approximately 5.8%. Therefore, the calculation results indicated that 500μm Be adopted as a substrate won't bring very many adverse effects to the X-ray intensity in this novel target.

### 2. Simulation of the Effects of *I*_*T*_ / *I*_*S*_ on the CT Image Resolution

**Fig 8:**
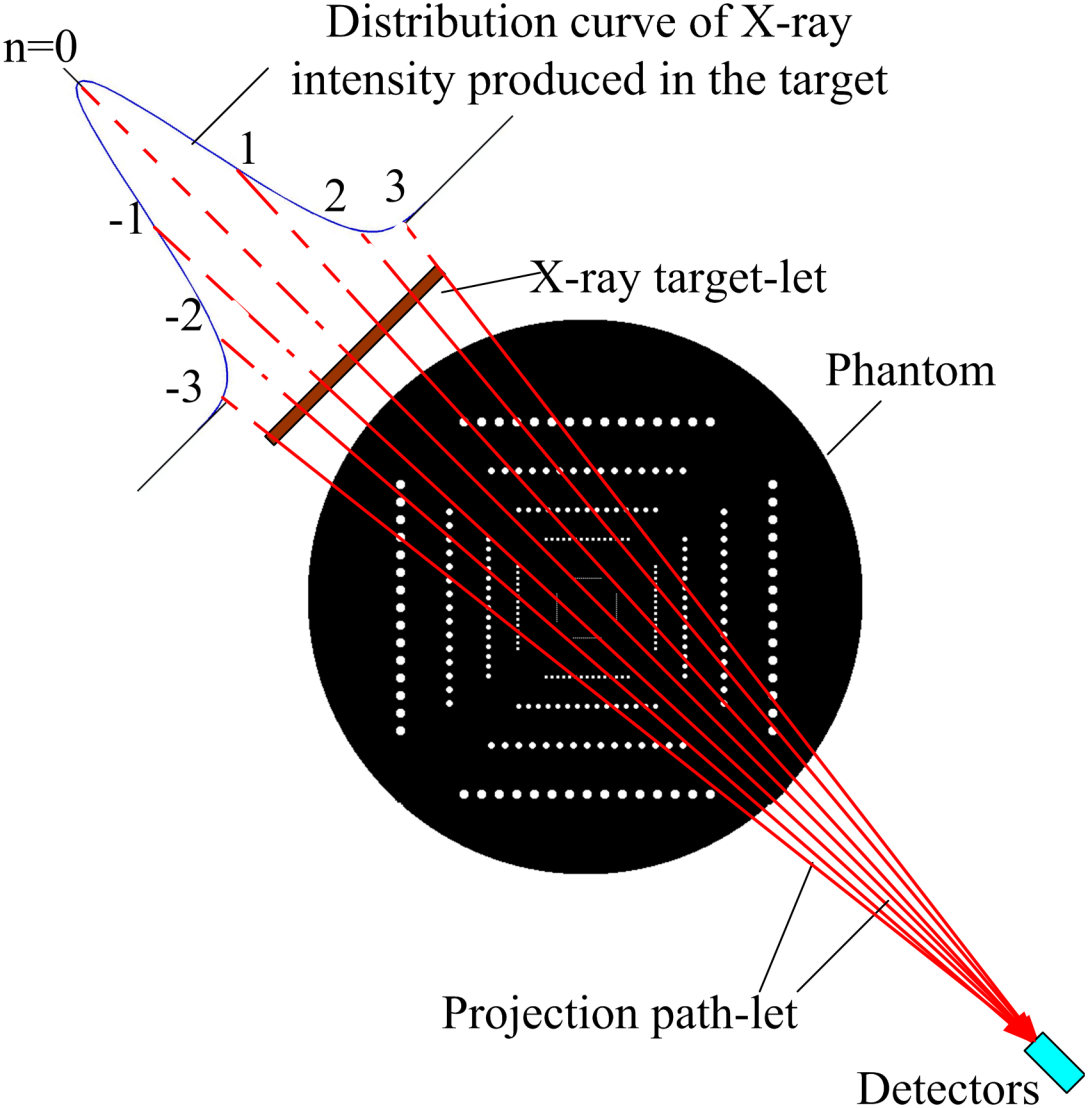
Simulation model of CT reconstruction used an X-ray source with a Gaussian distribution intensity, and the detector-to-ISO distance is 58mm, the source-to-ISO distance is 2.0mm, so the detector can be considered as an ideal point (i.e. so-called detector model) (To display more clearly, the X-ray target is enlarged disproportionately).

To discuss and investigate the effect of *I*_*T*_ / *I*_*S*_ on the CT image resolution, achieving a reasonable *I*_*T*_ / *I*_*S*_ for the point-like target design, a simplified simulation model is established as shown in Fig 8. Since the detector-to-object distance 58 mm is much more than the distance of the source-to-object 2.0 mm, the detector can be taken as an ideal point (i.e. so-called detector model) [25]. For the purpose of simple discussion, the X-ray intensity distribution of the photon fluence issuing from the micro-target is considered an approximate Gaussian distribution, and the X-ray beam is assumed to be monochromatic, defined as:

$$\Phi (\text{x})=\frac{1}{\sigma \sqrt{2\pi }}e^{-x^{2}/2\sigma^{2}}$$ (5)

where σ is the traditional parameter of the Gaussian function.In this model, the target is divided into *n* elements, and each element is called a target-let [25]. The length of the target is denoted as:

$$L_{t\text{arg}et}=n\cdot \Delta p$$ (6)

where *n* is generally the number of the target-lets, *n* = 7 is reasonable [25,26]. Δ*p* is the length of every pixel related to the phantom. So the discrete form of the (Eq 5) can be denoted as:

$$\Phi (n\Delta p)=\frac{1}{\sigma \sqrt{2\pi }}e^{-{\left(n\Delta p\right)}^{2}/2\sigma^{2}}$$ (7)

The projection path-lets of CT simulation for a detector are shown in Fig 8. The data Φ(*X*) received by the detector is:

$$\Phi (X)=\sum_{n=-3}^{n=3}\Phi (n\Delta p)e^{-\int u(\text{x},\text{y})dl}$$ (8)

where *μ*(*x*,*y*) are attenuation coefficients of X-ray (or image gray value).

**Fig 9:**
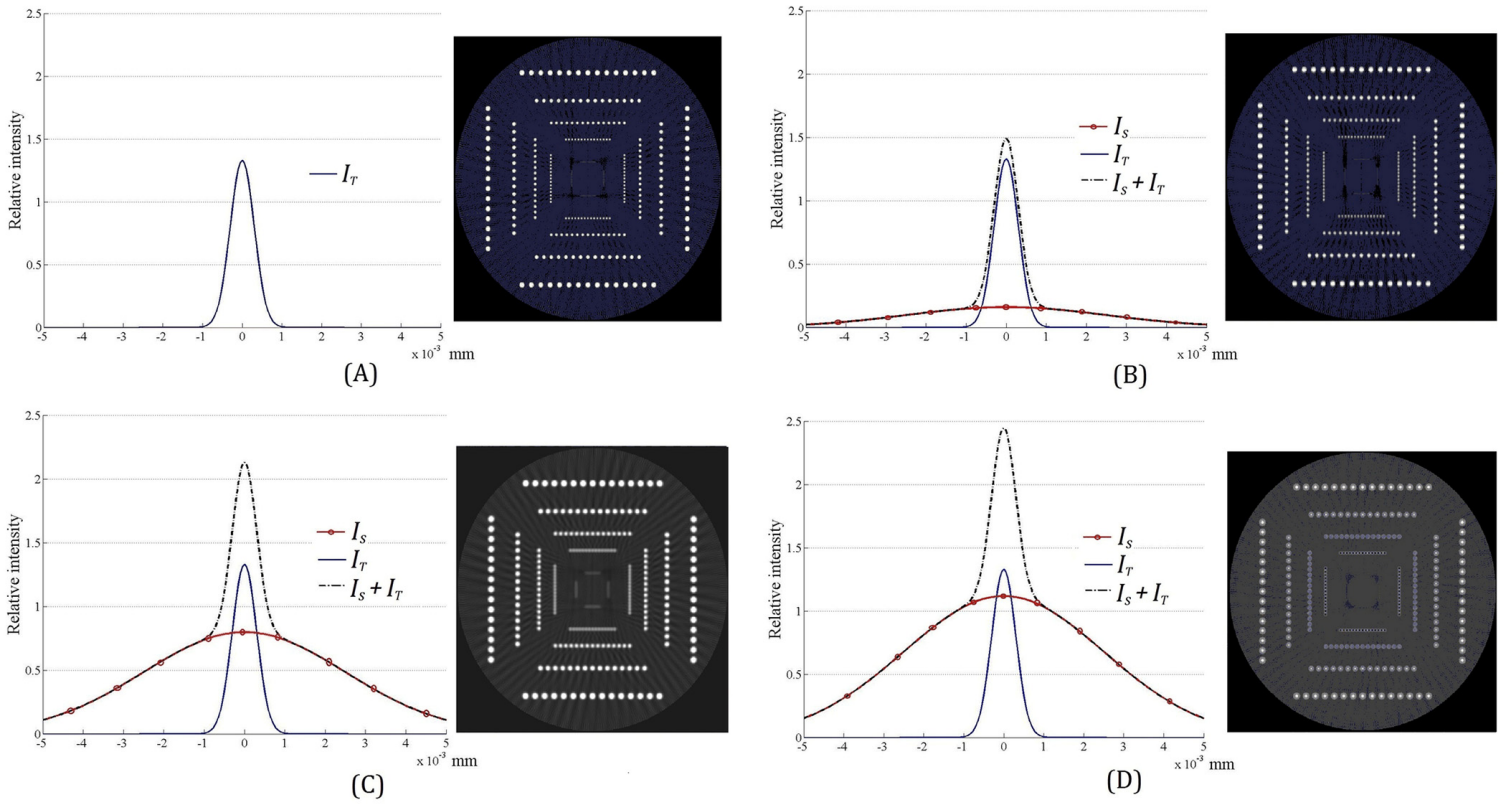
Effects of different *I*_*T*_ / *I*_*S*_ on the CT image resolution. (A) *I*_*S*_ = 0; (B) *I*_*T*_ / *I*_*S*_ = 2, degradation of CT resolution isn’t obvious; (C)*I*_*T*_ / *I*_*S*_ = 1/2, degradation of CT resolution is tolerable; (D) *I*_*T*_ / *I*_*S*_ = 1/5, CT resolution degrades seriously. (Simulation CT image is 512×512 pixels).

The CT simulation results are exhibited in Fig 9(A), 9(B), 9(C) and 9(D). When *I*_*T*_ / *I*_*S*_> 1/2, as shown in Fig 9(A) and 9(B), the CT resolution is almost unaffected. If *I*_*T*_ / *I*_*S*_ = 1/2, displayed in Fig 9(C), the degradation of CT resolution caused by *I*_*S*_ may be tolerable because the second rows of small holes are nearly distinguished. However, if *I*_*T*_ / *I*_*S*_<1/2, the second rows of small holes become obscure greatly as shown in Fig 9(D). The degraded CT resolution is considered unacceptable.

Above simulated results are readily comprehensible. As displayed in Fig 9(B), when *I*_*T*_ / *I*_*S*_ = 2, the FWHM (full width at half maximum) of the total X-ray intensity curve (i.e. *I*_*T*_ + *I*_*S*_), which is considered as the effective X-ray spot size in general, equals the FWHM of the X-ray intensity *I*_*T*_ curve approximately. When *I*_*T*_ / *I*_*S*_ is decreased, the FWHM of the *I*_*T*_ + *I*_*S*_ curve becomes more and more broadened. When *I*_*T*_ / *I*_*S*_<1/2, the change of the FWHM of the *I*_*T*_ + *I*_*S*_ curve caused by the X-ray’s intensity *I*_*S*_ could not be ignored for CT image resolution as shown in Fig 9(D). Therefore, if the point-like target is optimally designed, i.e *I*_*T*_ / *I*_*S*_>1/2, the X-ray focal spot would be determined largely by the X-rays produced from the point-like micro-target. Thus the size and stability of the X-ray focal spot in the mircofocus tube are mainly determined by the point-like micro-target rather than depending on the incident electron beam.

## Discussion and Conclusion

In summary, we have proposed a novel concept to develop a microfocus X-ray tube based on a point-like micro-target for the purpose of reducing the micro-CT image artifacts which resulted from the X-ray focal spot shifting and focal spot size changing in the mircofocus X-ray tube. The simulated results are demonstrated that if the parameter of the point-like micro-target and the target substrate is designed optimally—for instance, a 30~90keVelectron beam with *S*_*T*_ ≥ *S*_*e*_/13,a tungsten point-like micro-target with 1.0~5.0μm height—*I*_*T*_ / *I*_*S*_ will greater than1/2. In addition, the CT simulation reconstructed images revealed that when *I*_*T*_ / *I*_*S*_ >1/2, the adverse effect caused by the bremsstrahlung radiation generated in the target substrate could be neglected for the CT image resolution. Consequently, the X-ray’s effective spot size and stability are mainly determined by the properties of the point-like micro-target instead of by the electron beam in a conventional X-ray tube, which could, in theory, effectively alleviate the artifacts in CT images caused by the X-ray focal spot shifting and size changing.

## References

[1] Badea CT, Johnston SM, Qi Yi, Johnson GA. 4D micro-CT for cardiac and perfusion applications with view under sampling. *Phys*. *Med*. *Biol*. 2011;56: 3351–3369. 10.1088/0031-9155/56/11/011 21558587 PMC3180888

[2] Du LY, Joseph U, Nikolov HN, Pollmann SI, Lee TY, Holdsworth DW. A quality assurance phantom for the performance evaluation of volumetric micro-CT systems. *Phys*. *Med*. *Biol*. 2007; 52: 7087–7108 10.1088/0031-9155/52/23/021 18029995

[3] Manuel D, Denis VL, Bert M, Jan VB, Joris VA, Veerle C, Luc VH. Recent micro-CT scanner developments at UGCT.*Nuclear Instruments and Methods in Physics Research B*. 2014; 324: 35–40 10.1016/j.nimb.2013.10.051

[4] Mertens JCE, Williams JJ, Chawla N. Development of a lab-scale, high-resolution, tube generated X-ray computed tomography system for three dimensional (3D) materials characterization. *Materials Characterization* 2014; 92: 36–48 10.1016/j.matchar.2014.03.002

[5] Hames MH, Flynn MJ, Reimann DA. Measurement of Very Small(1–10 micron) X-ray Focal Spot Intensity Distributions, Medical Imaging Research Center, Henry Ford Hospital, Detroit, MI 1993.

[6] Salamon M, Hanke R, Kruger P, Sukowski F, Uhlmann N, Voland V. Comparison of different methods for determining the size of a focal spot of microfocus X-ray tubes. *Nuclear Instruments and Methods in Physics Research A* 2008;591: 54–58 10.1016/j.nima.2008.03.023

[7] Hiller J, Maisl M, Reindl LM. Physical characterization and performance evaluation of an x-ray micro-computed tomography system for dimensional metrology applications. *Meas*. *Sci*. *Technol*. 2012;23: 1–18. 10.1088/0957-0233/23/8/085404

[8] Vogeler F, Verheecke W, Voet A, Kruth JP, Dewulf W. Positional Stability of 2D X-ray Images for Computer Tomography. *Int* *Symposium on Digital Industrial Radiology and Computed Tomography* location:Berlin, date:20–22 June 2011.

[9] Kim JH, Nuyts J, Kyme A, Kuncic Z, Fulton R. A rigid motion correction method for helical computed tomography (CT).*Phys*. *Med*. *Biol*. 2015;60: 2047–2073. 10.1088/0031-9155/60/5/2047 25674780

[10] Yu HY, Wei YC, Hsieh J, Wang G. Data consistency based translational motion artifact reduction in fan-beam CT.*IEEE TRANSACTIONS ON MEDICAL IMAGING*.2006; 25: 792–803. 10.1109/TMI.2006.875424 16768243

[11] Ihsan A, Heo SH, Cho SO. A microfocus X-ray tube based on a microstructured X-ray target. *Nuclear Instruments and Methods in Physics Research B*. 2009; 267: 3566–3573. 10.1016/j.nimb.2009.08.012

[12] Hippler R, Saeed K, McGregor I, Kleinpoppen H. Z dependence of bremsstrahlung radiation from free atoms. *Phys*. *Rev*. *Lett*. 1981;46: 1622.

[13] Semaan M, Quarles C. Z dependence of atomic-field bremsstrahlung, *Phys*. *R*ev. A 1982; 26: 3152–3154. 10.1103/PhysRevA.26.3152

[14] Ebel H. X-ray tube spectra *X-Ray Spectrom* 1999; 28: 255–266.

[15] Kramers H. On the theory of X-ray absorption and the continuous X-ray spectrum, *Philosophical Magazine*.1923;46: 836–871.

[16] Ambrose V, Quarles C, Ambrose R. Thin-target bremsstrahlung at 0 degrees from 50 keV electrons, *Nucl*. *Instrum*. *Methods Phys*. *Res*. *B* 1997; 124: 457–463. 10.1016/S0168-583X(97)00099-2

[17] Trincavelli J, Castellano G. The prediction of thick target electron bremsstrahlung spectrain the 0.25–50 keV energy range. *Spectrochimica Acta Part B* 2008;63: 1–8. 10.1016/j.sab.2007.11.009

[18] Desobry GE, Boyer AL. Bremsstrahlung review: An analysis of the Schiiff spectrum. *Med*. *Phys*. 1991; 18: 497–505.19 1870494 10.1118/1.596653

[19] Monfared AS, Pourfallah TA, Babapour H, Shirazi AR. HVL evaluation of or thovoltage X-ray machine using EGSnrc code of simulation. International journal of radiation research. 2014; 12: 325–330.

[20] Aviles JEA, Pistorius S, Elbakri IA, Gordon R, Ahmad B. A 1st generation scatter CT algorithm for electron density breast imaging which accounts for bound incoherent, coherent and multiple scatter: A Monte Carlo study. *Journal of X-Ray Science and Technology*. 2011; 19: 477–499. 10.3233/XST-2011-0308 25214381

[21] Tavora LMN, Morton EJ, Gilboy WB. Enhancing the ratio of fluorescence to bremsstrahlung radiation in X-ray tube spectra *Applied Radiation and Isotopes* 2001; 54: 59–72. 10.1016/S0969-8043(00)00165-2 11144254

[22] Kawrakow, Rogers DWO. The EGSnrc Code System: Monte Carlo simulation of electron and photon transport Technical Report PIRS–701 (4th printing), National Research Council of Canada, Ottawa, Canada,2003.

[23] Treurniet JA,Walters BRB, Rogers DWO. BEAMnrc, DOSXYZnrc and BEAMDP GUI User’s Manual. NRC Report PIRS 0623(rev C) 2004.

[24] Tian LX, Zhu JJ, Liu MT, An Z. Bremsstrahlung spectra produced by kilovolt electron impact on thick targets *Nuclear Instruments and Methods in Physics ResearchB*2009;267: 3495–3499. 10.1016/j.nimb.2009.08.009

[25] Hsieh J. Computed Tomography—Principles, Design, Artifacts,and Recent Advances. Bellingham, Washington, USA: SPIEPress; 2003.

[26] Kalender WA. Computed Tomography: Fundamentals, SystemTechnology, Image Quality, Applications. Erlangen: PublicisCorporate Publishing; 2005.

